# C-reactive protein point-of-care testing and complementary strategies to improve antibiotic stewardship in children with acute respiratory infections in primary care

**DOI:** 10.3389/fped.2023.1221007

**Published:** 2023-10-12

**Authors:** Annamaria Staiano, Lars Bjerrum, Carl Llor, Hasse Melbye, Rogier Hopstaken, Ivan Gentile, Andreas Plate, Oliver van Hecke, Jan Y. Verbakel

**Affiliations:** ^1^ Department of Translational Medical Sciences, University of Naples “Federico II”, Naples, Italy; ^2^ Centre for General Practice, Department of Public Health, University of Copenhagen, Copenhagen, Denmark; ^3^ Department of Public Health and Primary Care, University of Southern Denmark, Odense, Denmark; ^4^ General Practice Research Unit, Department of Community Medicine, The Arctic University of Norway, Tromso, Norway; ^5^ Department of General Practice, CAPHRI School for Public Health and Primary Care, Maastricht University Medical Centre, Maastricht, Netherlands; ^6^ Section of Infectious Diseases, Department of Clinical Medicine and Surgery, University of Naples Federico II, Naples, Italy; ^7^ Institute of Primary Care, University and University Hospital Zurich, Zurich, Switzerland; ^8^ Nuffield Department of Primary Care Health Sciences, University of Oxford, Oxford, United Kingdom; ^9^ NIHR Community Healthcare Medtech and IVD Cooperative, Nuffield Department of Primary Care Health Sciences, University of Oxford, Oxford, United Kingdom; ^10^ EPI-Centre, Department of Public Health and Primary Care, Academisch Centrum Voor Huisartsgeneeskunde, Leuven & NIHR Community Healthcare Medtech and IVD cooperative, Leuven, Belgium

**Keywords:** c-reactive protein, antibiotic prescribing, antibiotic stewardship, antimicrobial resistance, point-of-care testing, respiratory tract infections, children

## Abstract

This paper provides the perspective of an international group of experts on the role of C-reactive protein (CRP) point-of-care testing (POCT) and complementary strategies such as enhanced communication skills training and delayed prescribing to improve antibiotic stewardship in the primary care of children presenting with an acute illness episode due to an acute respiratory tract infection (ARTI). To improve antibiotics prescribing decisions, CRP POCT should be considered to complement the clinical assessment of children (6 months to 14 years) presenting with an ARTI in a primary care setting. CRP POCT can help decide whether a serious infection can be ruled out, before deciding on further treatments or management, when clinical assessment is unconclusive. Based on the evidence currently available, a CRP value can be a valuable support for clinical reasoning and facilitate communication with patients and parents, but the clinical assessment should prevail when making a therapy or referral decision. Nearly half of children tested in the primary care setting can be expected to have a CRP value below 20 mg/l, in which case it is strongly suggested to avoid prescribing antibiotics when the clinical assessment supports ruling out a severe infection. For children with CRP values greater than or equal to 20 mg/l, additional measures such as additional diagnostic tests, observation time, re-assessment by a senior decision-maker, and specialty referrals, should be considered.

## 1. Introduction

### 1.1. Antimicrobial resistance

Bacterial antimicrobial resistance (AMR) is flagged as one of the top 10 threats to global health by the World Health Organization (1). Although it has often been referred to as the “silent pandemic” (2) because its effects are not felt at the place and time they are caused, AMR has been a topic of enduring discussion for many years. The effects of AMR are already drastic today and far from “silent”, causing 1 of 3 hospital deaths due to untreatable secondary bacterial infections (3), and in 2019 a total of 1.27 million deaths globally directly attributable to bacterial AMR, and estimated deaths associated with bacterial AMR at 4.95 million (3). The effects of AMR will be felt even more broadly and severely by our future generations, with 10 million deaths expected globally due to drug-resistant diseases by 2050 (6), with extended impacts on economic stability and increased poverty as early as 2030 (4).

Today's children, if society fails to act and use antibiotics more responsibly, may not have effective treatment for infections that today are cured with a week of antibiotic treatment, essentially catapulting treatment options back 80 years due to the obsolescence of current antibiotics. Already today, AMR is flagged as a threat to neonate survival (5). An effective stewardship of antibiotics could help ensure that the essential life-saving treatment that antibiotics provide remains effective for future generations. That means creating awareness, convictions, and behaviors and supporting mechanisms that ensure antibiotics are used effectively and in a more targeted way. The primary care setting is ideal for children and parents alike to learn about good antibiotic stewardship behaviors, but more importantly a vital setting to influence antibiotic prescribing behaviors, as a considerable portion of antibiotic prescribing is attributable to the primary care of children presenting with an ARTI (6). A recent systematic review and meta-analysis including 86 studies comprising more than 11 million children, concluded a pooled antibiotic prescribing rate of 45.4% for all acutely ill children (7). About one-fifth to one-half of these antibiotic prescriptions are estimated to be inappropriate (7). In most cases of chest infections, particularly when there is no suspected pneumonia, clinicians should not prescribe antibiotics but instead provide safety netting advice. A safety net advice can be defined as an advice about what to do and what to look out for, empowering parents and carers to seek help if the child's condition deteriorates further or if they need more support (8).

### 1.2. C-reactive protein

CRP is a non-specific inflammatory marker. CRP values correlate well, with the degree of inflammation in the body without identifying the specific cause (9). It raises rapidly (within 4–6 h), but it may take 24–48 h to reach maximum. Indeed, CRP levels can be raised due to auto-immune conditions, cancers, and infections, and therefore cannot with certainty identify if an inflammation is caused by a virus, bacteria, or other. On the other hand, there are documented cases where children's CRP levels remain low despite serious bacterial infections (10, 11). Together with an assessment of other clinical parameters, however, the CRP level is an important predictor of severe ARTI (pneumonia, sepsis) (12). But its main power is, particularly in primary care, to support whether an infection is mild and self-limiting or not. Self-limiting infections are those that tend to resolve themselves without further treatment and represent most respiratory tract infections. If an inflammation is not severe, and appears to be self-limiting, antibiotic treatment will not be beneficial and may even be harmful, causing side effects, alterations to the microbiome (13) and eventually contribute to AMR. If a respiratory tract infection is severe, clinicians should consider starting antibiotic treatment or even urgently refer to a hospital to avoid aggravation or complications, like sepsis. CRP values offer a reliable indicator to complement a general clinical assessment to decide on management. There are opportunities to decrease antibiotic over-consumption by leveraging CRP POCT, when combined with clear guidance and effective communication strategies, for the treatment of children presenting with an ARTI.

## 2. Evaluation of evidence supporting CRP POCT effectiveness in safely reducing antibiotic prescribing for children presenting with an ARTIs

The use of quantitative CRP POCT to reduce antibiotic prescribing for adults in the primary care settings has been well evaluated, and there is ample evidence indicating a high effectiveness of the tests in safely reducing antibiotic prescribing, especially for adult patients presenting with symptoms of LRTIs (14–25). In adults with LRTIs, up to 42% antibiotic prescribing rate reductions were observed (42% relative reduction; 22% absolute reduction, 31% vs. 53%) (19), and more even greater reductions were measured when CRP POCT was combined with targeted communication skills training (19, 20).

While the volume of data regarding the effectiveness of CRP POCT in safely reducing antibiotic prescribing for children is not as complete, an evaluation of current clinical evidence shows a clear opportunity for use in primary care. As in the case of adults presenting to primary care, broader adaptation of CRP POCT to complement the clinical assessment of children presenting in primary care with an ARTI could contribute to safely reducing antibiotic prescribing rates, especially when clear guidance on when to perform CRP POCT and effective communication strategies for clinicians are provided.

The role of CRP in reducing antibiotic prescription in the primary care of children presenting symptoms of respiratory tract infections has been under evaluation for some time, but results have not always been conclusive enough to make clear recommendations (21–23). More recent systematic reviews indicate a clear potential to safely reduce antibiotic prescribing for children with ARTIs:

• In 2019, Verbakel et al. and in 2020 Martinez-Gonzales et al. calculated an antibiotic prescribing reduction rate of 44% for children in randomized studies once CRP cut-off guidance was applied, and this without any negative effect on patient outcomes or healthcare processes (RR 0.56, 95% CI 0.33–0.95) (15, 16).
• In 2020, Van Hecke et al. concluded that there is emerging evidence that CRP POCT can be effective at reducing antibiotic prescribing for children with acute RTIs in lower and middle-income countries, but that evidence of the effectiveness in high-income countries is not as abundant (24).
• In the updated systematic review on the impact of the use of CRP on antibiotic prescribing by Smedemark et al. in 2022, a total of four randomized clinical trials including 2,335 children (Althaus 2019; Diederichsen 2000; Do 2016; Schot 2018), collectively found that CRP rapid testing reduces the number of children given an antibiotic prescription by 22%, with this effect being primarily seen in low- and middle-income countries (0.78, 95% CI 0.67–0.91) (25).

The table below summarizes several studies performed in primary care and in emergency department settings related to the effectiveness of CRP POCT in children to safely reduce antibiotic prescribing rates or rule in or out serious infection. The search strategy is detailed in the Appendix.

Overall, it would be useful to generate more context-specific evidence to better understand best practices in utilizing CRP POCT to reduce antibiotic prescribing and consumption in pediatric primary care settings worldwide (15). That said, the authors feel confident that the below guidance, based on the information available today, will allow for a safe and significant contribution to antibiotic stewardship.

## 3. The use of CRP POCT in the primary care of children presenting with an ARTI

When a physician is deciding to prescribe antibiotics, it may be relevant to perform CRP POCT to support the assumption that those antibiotics will be beneficial and avoid over-prescription. This can help to prevent prescriptions given to worried parents based on “gut feeling” or motivated by a “better safe than sorry” approach and will reassure both the physician and the parent that the right choice has been made.

CRP POCT can help to reduce diagnostic uncertainty and can serve as a way to support communication with parents when antibiotics are not advised. This can be especially useful when parents and children have difficulties in describing the severity of symptoms, or parents are worried and need to be reassured.

A CRP POCT can also support the decision not to prescribe antibiotics, especially when there is any degree of uncertainty. In cases of severe (bacterial) infections, it is important to start antibiotic treatment quickly to avoid exacerbation, complications, and prolonged discomfort. A CRP POCT can be an important component of safety netting procedures to support the decision not to prescribe antibiotics.

Additionally, CRP values can be used to communicate and demonstrate that antibiotic treatment is not recommended to a concerned parent that may have been pushing for or expecting antibiotic treatment. This argumentation value can be especially pertinent for regions where antibiotic use is common-place and access to antibiotics is possible without a prescription.

While using a CRP POCT is useful to complete the clinical assessment of children presenting with ARTIs, the decision whether to perform a test and the ultimate treatment is always at the discretion of the treating physician, considering all clinical and non-clinical factors.

## 4. Guidance on the interpretation of CRP results for the primary care of children presenting with an ARTI

Simply having a CRP value is not enough to reduce antibiotic prescribing. Clear guidance is key to achieving safe and meaningful reductions. Guidance supports physicians treating children with RTIs in their decision making in order to safely and confidently reduce overall antibiotic prescribing rates, while ensuring that antibiotics are prescribed, or a hospital referral takes place, when needed. However, any guidance must always be interpreted in light of a complete clinical assessment.

**Figure 1:**
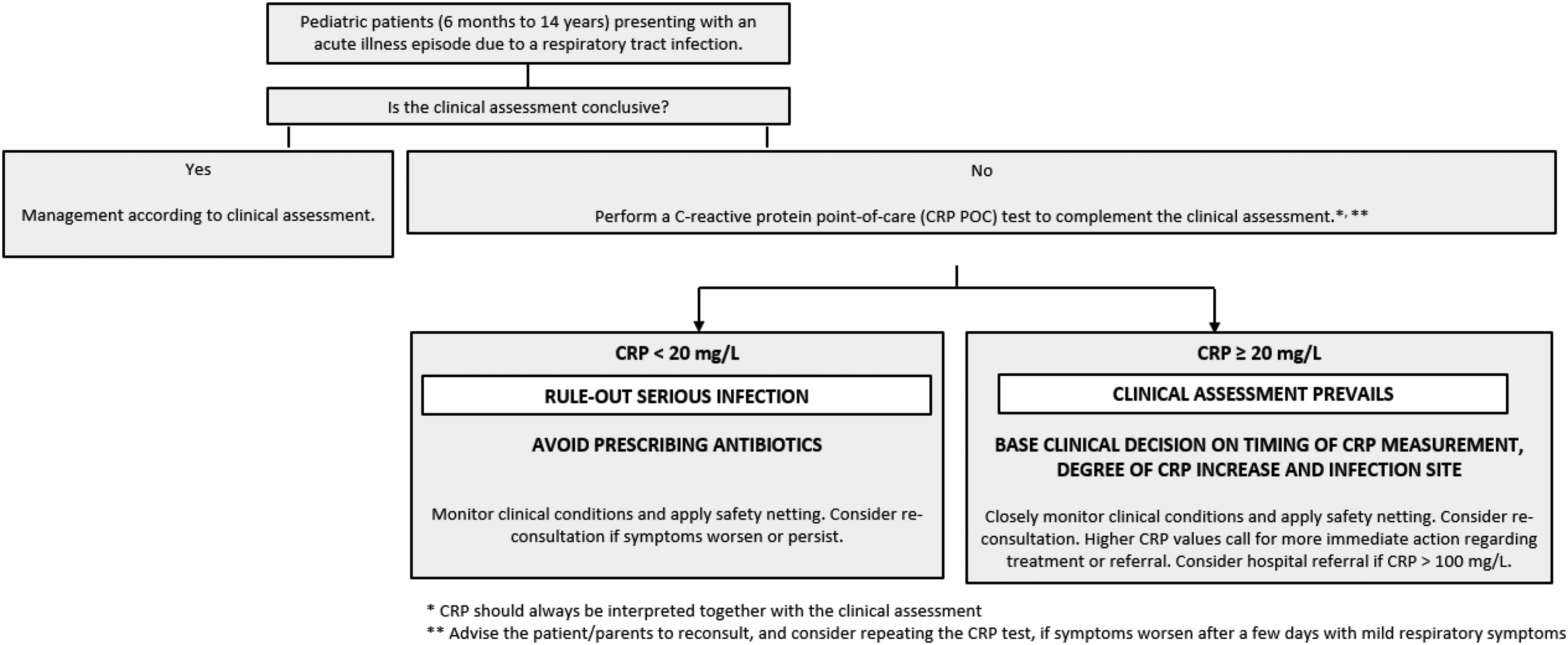
Decision tree/flowchart including the interpretation of CRP values for pediatric population (from 6 months to 14 years) presenting with an acute illness episode due to a respiratory tract infection in primary care.

Figure 1 offers guidance on the interpretation of CRP values for patients from 6 months to 14 years presenting with an ARTIs, based on the synthesis of several research results and publications—see Table 1 and recent systematic reviews and meta-analysis (25). The suggested cut-offs must be interpreted in the context of a complete clinical assessment.

**Table 1:** Overview of studies examining the role of CRP POCT in safely reducing antibiotic prescribing rates for children with RTIs.

Paediatrics primary care				
First author/journal/year of publication	Design/study type	Indications/population/symptoms	Guidance CRP values Cut-off values	Results and conclusions
Keitel, Clinical infectious diseases: an official publication of the Infectious Diseases Society of America 2019 (26)	RCT 1,726 children (2 months—4,9 years)	Children with fever and cough and without life-threatening conditions	Guidance: Antibiotics, if combination of CRP ≥80 mg/l plus age/temperature-corrected tachypnea and/or chest indrawing or current respiratory rate ≥50 breaths/minute (World Health Organization standard)	CRP testing using a cutoff of ≥80 mg/l, integrated into an electronic decision algorithm, was able to improve clinical outcome in children with respiratory infections while substantially reducing antibiotic prescription in primary care centers in Tanzania. 2.3% children in the intervention arm vs. 40.4% in the control arm received antibiotics at day 0 (RR, 0.06). There were fewer secondary hospitalizations and deaths in the CRP arm: 0.5% vs. 1.5% (RR, 0.30).
Althaus T, The Lancet Global health 2019 (27)	Randomized study 2,410 patients (1,201 aged 1–11 years)	Documented fever or a chief complaint of fever (75–82% with respiratory symptoms depending on age groups)	Guidance: 2 CRP testing groups; antibiotic prescription if:	In febrile patients attending primary care, testing for CRP at point of care with a threshold of 40 mg/l resulted in a modest but significant reduction in antibiotic prescribing, with patients with high CRP being more likely to be prescribed an antibiotic, and no evidence of a difference in clinical outcomes. Antibiotic prescribing from 0 up to 5 days: - Control group 39% - CRP ≥40 mg/l: 34%, sign. OR 0,8 - CRP ≥20 mg/l: 36%, non-sign. OR 0,86 Patients presenting with a respiratory syndrome in both intervention groups showed a significant reduction in antibiotic prescription.
			- ≥20 mg/l (group A) ≥40 mg/l CRP (group B)	
Schot, BJGP Open 2018 (22)	RCT	Children with acute cough and fever	Guidance:	Antibiotic prescribing was lower in the CRP group (30.9%) versus the control group (39.4%), but the difference was not statistically significant (OR 0.6).
	301 children	1. POC CRP levels should be interpreted in combination with symptoms and signs. 2. CRP <10 mg/l make pneumonia less likely, but should not be used to exclude pneumonia when the GP finds the child ill, or when duration of symptoms is <6 h. 3. CRP >100 mg/l make pneumonia much more likely, however, such levels can also be caused by viral infections. 4 Between 10 mg/l and 100 mg/l, the likelihood of pneumonia increases with increasing CRP levels.	This may in part be due to lack of power to detect a smaller than expected decrease. Antibiotic	
	(3 months—12 years)		Prescribing rates were lower than expected. 46% of children had POC CRP levels <10 mg/l.	
Higdon, Clin Inf Dis 2017 (28)	Observational	601 HIV-negative children with RTI <5years	No fixed cut-offs	Among cases with confirmed bacterial pneumonia, 77% had CRP ≥40 mg/l compared with 17% of 556 RSV pneumonia cases. The Receiver Operating Curve (ROC) analysis produced an AUC of 0.87, indicating very good discrimination; a cut-point of 37.1 mg/l best discriminated bacterial pneumonia (sensitivity 77%) from RSV (specificity 82%).
Rebnord, BMJ Open 2017 (29)	RCT	401 children with RTI or fever 0–6 years	No fixed cut-offs	CRP values >20 mg/l, findings on ear examination, use of paracetamol and no vomiting in the past 24 h were significantly associated with antibiotic prescription.
Do NT, Ta NT, Lancet Glob Health 2016 (30)	RCT 1,028 children (1–15 years) and 1,009 adults (16–65 years)	Acute RTIs Symptoms at presentation were: • Cough (88/89%), • Sore throat (82/82%), • Coryza (62/61%), • Fever (36/34%), • Dyspnoea (2/3%), and earache (5/4%) for the CRP/control group.	Guidance: No antibiotics if • CRP ≤ 10 mg/l for patients aged 1–5 years • CRP ≤ 20 mg/l for patients aged 6–65 years Generally, antibiotics and consideration of hospital referral if • CRP ≥ 50 mg/l for patients aged 1–5 years • CRP ≥ 100 mg/l for patients aged 6–65 years	CRP POCT reduced antibiotic use for non-severe acute respiratory tract infections without compromising patients’ recovery in primary health care in Vietnam. Patients receiving any antibiotics within 14 days of follow-up: - Children (1–15 years) OR 0,55 (*p* 0.0001) - Adults (>15 years) OR 0,41)*p* < 0.0001)
Diez-Padrisa, Trop Med 2012 (31)	Observational	586 African hospitalized children with clinical severe pneumonia <5years	No fixed cut-offs	A cut-off of 38 mg/l was associated with bacteremia with the most reasonable sensitivity/specificity values
Diederichsen, SJPHC 2000 (32)	RCT (POC vs. no intervention)	812 adults and children with RTI 0–90 years	No fixed cut-offs	No reduction in antibiotic prescription, but CRP values in the POC group was the most relevant factor for antibiotic prescription
Paediatrics Emergency Department				
Marcus N, European Journal of Emergency Medicine 2008 (33)	Prospective study	55 Children with pneumonia	Results: Mean CRP levels • Bacterial pneumonia: 121.3 +/- 122 mg/l viral pneumonia: 27.2 ± 26 mg/l (*P* = 0.007) • In the patients who presented before 96 h of symptom onset	The CRP test seems to be an useful predictor of bacterial pneumonia in children, especially those with a shorter illness duration, and is feasible for use in the ED. With CRP: Area Under the Curve (AUC) 0,79.

The following treatment considerations are suggested for the CRP ranges listed below, for the primary care of children presenting with an acute illness episode due to a respiratory tract infection:

### 4.1. If the clinical assessment is non-conclusive, test CRP. If CRP <20 mg/L, avoid prescribing antibiotics

Nearly half of the children tested in the primary care setting for LRTIs will have a CRP below 20 mg/L, showing a considerable potential to rule out the need for antibiotic prescription (10, 22, 34, 35).

In these cases, it is strongly suggested to avoid prescribing antibiotics when the clinical assessment rules out a severe infection. Instead, clinicians should give safety netting advice and ask the child's guardian to monitor the progression of symptoms and in case of exacerbation or persistence to re-contact the physician. Re-consultation should be considered as an option especially in cases where the child presented so early in the progression of the disease (i.e.,: in the first 24 h) that a conclusive clinical evaluation and interpretation of CRP values is difficult or impossible. Consider prescribing symptomatic treatment. In case of worsening of the clinical conditions, clinicians should consider repeating CRP testing, taking into account the possibility of a secondary bacterial infection.

### 4.2. CRP ≥20 mg/L: the clinical assessment prevails, and CRP values can be used to complement clinical reasoning

For children with CRP values greater than or equal to 20 mg/L, the clinical assessment of the patient prevails, and CRP values can be used to complement clinical reasoning.

• The timing of the CRP measurement with regards to disease progression should be considered.
  ◦ CRP values are less reliable as predictive indicators during the first 24 h after the onset of a disease (36), and serious (life-threatening) infections such as sepsis can develop within this time.
  ◦ In the primary care of children, early presentation is common, and in these cases any interpretation of CRP results should be considered carefully and the progression of clinical conditions should be closely monitored. In general, the CRP threshold of 5 mg/L is often exceeded within four to eight hours after an acute inflammatory event, with CRP values peaking at 20–500 mg/L after 48 h (37, 38).
  ◦ Monitoring CRP over time can help differentiate serious infections from uncomplicated RTIs. If high CRP levels persist during the course of illness, further diagnostic workup should be considered to rule out serious infections.
• The degree of elevation of CRP values should be considered
  ◦ Higher CRP values indicate a greater urgency to act with regards to starting a course of treatment or referring to a hospital.
  ◦ Elevated CRP levels do not necessarily mean that antibiotics are a useful course of action, as inflammatory conditions and several viral infections can also increase CRP values in children (39). Be aware that a maximum CRP response with values above 40 mg/L is not infrequently found on days 3–4 in uncomplicated viral RTI (36).
  ◦ In cases of slightly elevated CRP values that exceed 20 mg/L with few clinical indications of a severe infection, clinicians may consider providing safety netting advice and prescribing symptomatic treatment for a specified period, with potentially a re-consultation if practically possible in case of exacerbation or persistence of symptoms.
  ◦ In cases of high CRP values (i.e.,: >75 mg/L) it is strongly suggested to start treatment with antibiotics, due to a high risk of a non-self-limiting infection, or to refer to a hospital (10). The child's guardians should monitor for an improvement of symptoms within a specified period of time, and if symptoms persist to arrange a re-consultation or present to a hospital.
  ◦ CRP levels exceeding 100 mg/L indicate a severe infection for which urgent referral to a hospital should at least be considered alongside thorough clinical assessment and history taking.
• The suspected type of infection plays an important role when making a therapy decision.
  ◦ Although several studies have found an increase in CRP levels with the severity of otitis media, the use of CRP levels as a guide for antibiotic prescribing in children with suspected ear infections has not specifically been investigated (40, 41). Most children with ARTI in primary care and with CRP ≥ 20 mg/L have viral infections, in which case antibiotics are not recommended. For children under 1 year of age, symptoms of lower RTIs are often associated with viral infections such as the RS-virus, for which antibiotic treatment is not recommended and hospital referral may be necessary (42).
  ◦ For cases where pneumonia is suspected, consider prescribing antibiotics when CRP values exceed 40 mg/L (43).

While these ranges are broadly applicable, the importance of an individual evaluation of each patient is key and should be leading the final treatment decision.

## 5. Complementary communication strategies

Both physicians and parents, and one day the children that are being treated, need to play a role as antibiotic stewards. The consultation is a key moment and great opportunity for physicians to offer parents and patients to be partners in the decision-making process. The consultation is typically a moment when symptoms are acute, making the messaging around antibiotic usage relevant and timely. Here below a few communication strategies to have impactful interactions that can educate and sensibilize, but also to put parents at ease with the treatment decision (especially important when access to antibiotics is possible without prescriptions) and potentially to increase satisfaction with regards to the parent/patient-physician interaction.

### 5.1. Announcement approach

The announcement approach simply states that physicians should give a clear and strong message to the patients, recommending a course of action. While there is no specific evidence regarding the impact of the announcement method on antibiotic use, research has shown that the words of a treating physician carry significant weight in influencing patient and parent acceptance of treatment advice. A study on HPV vaccination rates in the pediatric primary care setting showed that vaccination rates increased by 5% in 3 months by training the vaccine providers on making presumptive announcements (44).

In cases where the clinical evaluation of a physician leads to the decision not to prescribe antibiotic treatment, simple and clear statements like the following could be impactful.

- “Based on my current assessment, your child should not take antibiotics.”
- “We should not start an antibiotic treatment; it could do more harm than good.”

Naturally, there should be a follow-up conversation and further clarification provided where parents or patients express doubts or concerns.

### 5.2. Decision aids

If a decision aid is available, this could be reviewed together with a parent either in an explanatory fashion, or to engage them in a shared decision-making process. This process would not take much additional consultation time [on average 2.6 min (45)], but could increase the parent's knowledge, understanding and engagement with the antibiotic treatment decision—especially key to increase compliance in areas with easy access to antibiotics. Research has shown that using decision aids does not negatively impact patient satisfaction or health outcomes, and in the case of a decision aid to rule out pneumonia by patients presenting with cough and fever, had the potential to reduce antibiotic prescription by 9,1% (46).

### 5.3. Communication skills training

While potentially less straightforward to apply than the announcement approach, patient-centric consultation, and shared decision-making (physician-patient) techniques can further increase the effectiveness of a patient/parent-physician interaction. For instance, for adults presenting symptoms of LRTIs the use of CRP POCT reduces antibiotic prescribing by approx. 42% (relative reduction; 22% absolute reduction, 31% vs. 53%) (19), but combining CRP POCT with communication skills training reduces antibiotics prescribing reductions of more than 60% [absolute reduction 44%, 23% vs. 67% (19); RR 0.38; 95% CI 0.36–0.55 (20)]. While there is currently no clinical study available to confirm this, similar increases in efficacy can be expected for the pediatric setting.

During **patient-centric consultation** the physician takes a more individualized approach to patient care, emphasizing respect for individual preferences and empowering the patient. This involves asking not only for patient conditions and symptoms, but also concerns, perceptions, cultural influences, and taking these into account during treatment consideration.

When applying (physician-patient) **shared decision-making techniques**, the aim is to involve the patient more actively in the decision-making process, leading to a mutual decision in the best interest of the patient. Best practice combines evidence-based information with the clinical evaluation and experience of the physician, but also the patient's culture, values, and individual preferences.

### 5.4. Physician-parent interactions that leverage several effective strategies

Trust between physicians and parents is essential. A part of the trust is inherent to a physician's expertise, but a large part of trust is built on an inter-personal relationship based on strong communication. While physicians know how to speak with their patients' parents, and how to explain things to children, it can be useful to have a blueprint for approaches and key messages, especially in a context where the goal is to create a behavioral change that would lead to reduced antibiotic consumption.

**Figure 2:**
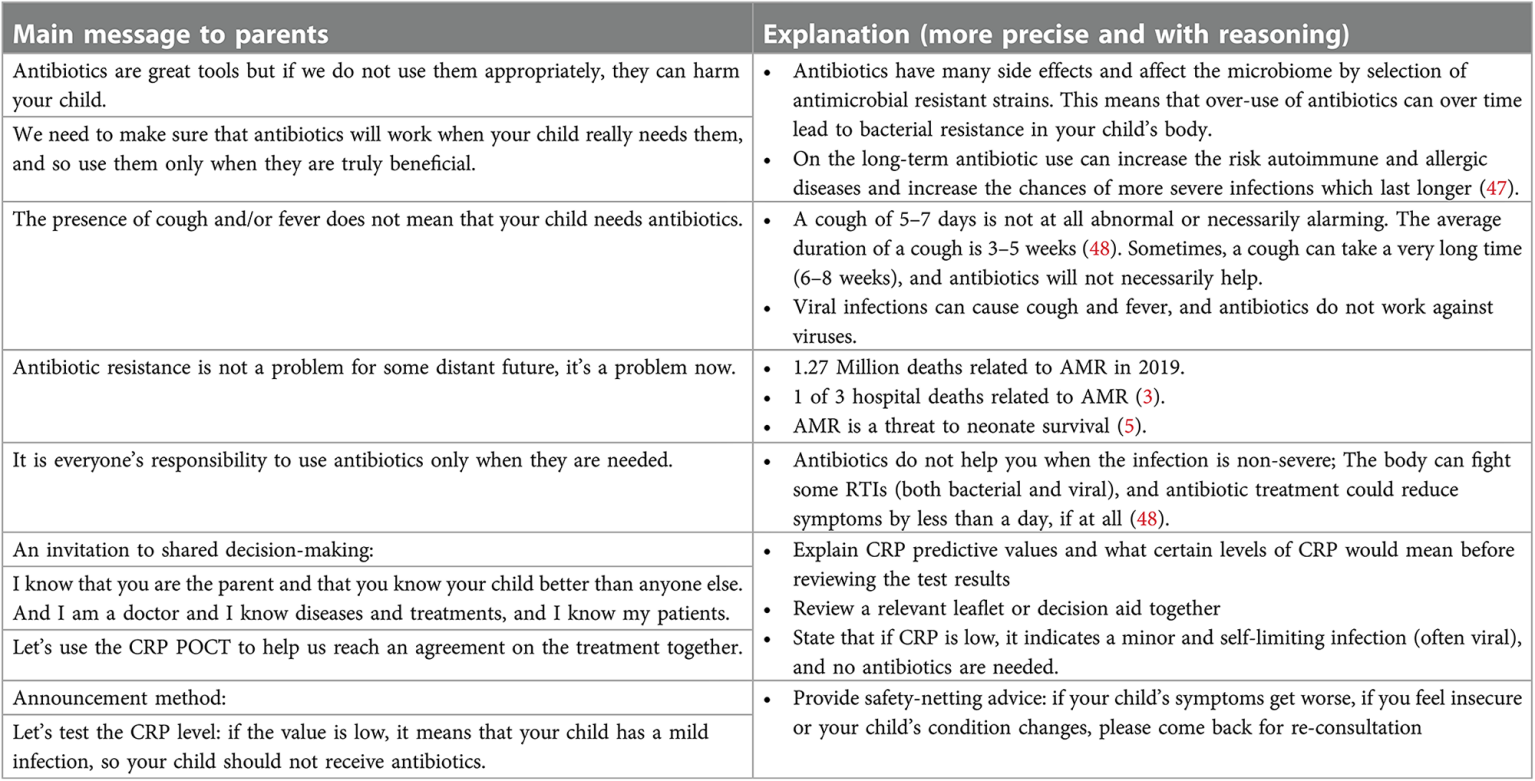
Example key messages and supporting facts.

Below (Figure 2) are a few key messages and supporting facts that could be transmitted during a consultation. These are not a script, but a number of talking points that could be useful, depending on the parent and patient the physician is facing. It may be pertinent, before introducing too many facts or arguments, to ask an open question such as: “What do you think of antibiotics?” or “To what extent do you think antibiotics would help your child?”. This would allow a physician to gauge the current attitude towards, desire for, and understanding of antibiotics and adjust the messaging accordingly.

## 6. Discussion

Based on the available literature, discussions between experts, and the available experience with CRP in adults, the authors recommend using CRP POCT in the primary care of children presenting with ARTIs, to safely reduce antibiotic prescribing rates. While the authors strongly suggest avoiding prescribing antibiotics when CRP values are below 20 mg/L and when the clinical assessment supports ruling out a severe infection, the degree of uncertainty, when it comes to the right cut-off values of CRP for children, is larger than in adults. That is why the most important evidence has been made available to the reader, and why the recommendations are always accompanied by the reminder that the physicians’ clinical examination and reasoning is paramount. For children CRP, irrespective of the CRP value, can be used to complement clinical reasoning.

### 6.1. The power and precautions surrounding rapid testing at the point-of-care

Having a point-of-care test available in the primary care setting drastically reduces or even eliminates waiting for the test result. This means physicians can immediately complete their clinical analysis with an objective test value and decide a course of action with more security. Patients and their families are not left waiting with doubt and worry, but can be given objective and convincing arguments about a course of action. This will improve the patient outcomes and experiences and improve adherence to the treatment decision. In addition, a CRP POCT can typically be performed by a nurse or medical assistant without requiring training in clinical laboratory sciences (15, 49, 50), allowing diversification of job content, and freeing up time for physicians to spend with patients.

POCT is a process of various elements that need to be considered to reach continuous high quality and to secure patient safety. It is advised to collaborate with POCT or laboratory experts for a proper start of POCT in primary care, and for the ongoing high quality of POCT in routine practice. This includes a workflow according to international standards with a standard operating procedure, proper user instructions at the start, and repeated training on testing performance at the testing site. Furthermore, a set of quality assurance aspects, including technical management of testing devices, tests, and controls, and feedback on technical and user errors, and proper communication of the test results with privacy-proof personal data (preferably within an electronical, closed circle) are recommended. This will increase the chance of ongoing high-quality and patient-safe use of POCT in primary care. To ensure the quality of CRP POCT please note that proper training specific to the CRP test needs to be provided.

### 6.2. The perils of certainty

As stated before, the treating physician always has the final decision regarding whether or not a CRP test would be a useful addition to their clinical assessment. Sometimes a physician may feel that indications are clear, and a CRP POC test is not needed. A certain degree of confidence is healthy and normal, but there are perils to certainty.

On the one hand, Physicians are generally confident in their antibiotic prescribing decision, with physicians rating 88% of their prescribing decisions at a level of confidence of certain or very certain (51). On the other hand, research has shown that close to half of antibiotics prescribed are not indicated by guidelines or considered over-prescription (47, 52). This underlines the disconnect between confidence levels and appropriateness of prescription. Therefore, it could be recommended to perform a CRP POC test to support and especially re-evaluate the antibiotic prescribing decision regardless of the physician's level of confidence, and to regard unexpected outcomes as learning opportunities that will contribute to better antibiotic prescribing behavior (53) and antibiotic stewardship.

## 7. Conclusion

To say that antimicrobial resistance is a threat is almost an understatement, with damaging impacts and high costs to both healthcare systems and individual patients already today. Action must be taken on several fronts to reduce the over-use of antibiotics and increase maturity of antibiotic stewardship, to limit antimicrobial resistance and keep the life-saving treatment that antibiotics provide effective for future generations. C-reactive protein point-of-care testing complemented with clear clinical guidance and effective communication techniques leads to better antibiotic stewardship, including a vast reduction of unneeded antibiotic prescriptions. It has the potential to trigger the behavioral change that is needed worldwide, especially with regards to high antibiotic prescribing settings for children presenting in primary care with an acute illness episode due to a respiratory tract infection. CRP POCT in combination with enhanced communication techniques and delayed prescribing should be considered to compliment the clinical assessment of children (6 months to 14 years) presenting with an ARTI in a primary care setting, in order to safely improve the appropriateness of antibiotic prescribing. When CRP values are below 20 mg/L and when the clinical assessment supports ruling out a severe infection, it is strongly suggested to avoid prescribing antibiotics. When CRP values are greater than or equal to 20 mg/L, CRP values can be used to complement clinical reasoning and further treatment considerations.

Further well-designed randomized controlled trials in children are needed to increase the evidence base, particularly on the best cut-off values of CRP in children for management decisions.

## Data Availability

The original contributions presented in the study are included in the article/Supplementary Material, further inquiries can be directed to the corresponding author.

## Appendix

We focused on articles included in the last Cochrane reviews. Additionally, we searched the most recent manuscripts using the PubMed database and the following keywords: CRP point of care or CRP AND Acute respiratory tract infections OR Respiratory tract infections AND children.
